# Supplementary material for: Novel Driver Strength Index highlights important cancer genes in TCGA PanCanAtlas patients
Source: PeerJ. 2022 Aug 11;10:e13860. doi: 10.7717/peerj.13860 (PMC9375969; doi:10.7717/peerj.13860)
Supplement: Supplemental Information 2 [file peerj-10-13860-s002.zip › COHORTS/genes plots/cohorts/2021_8_16_14_9_distribution_cohorts_CHOL.pdf]

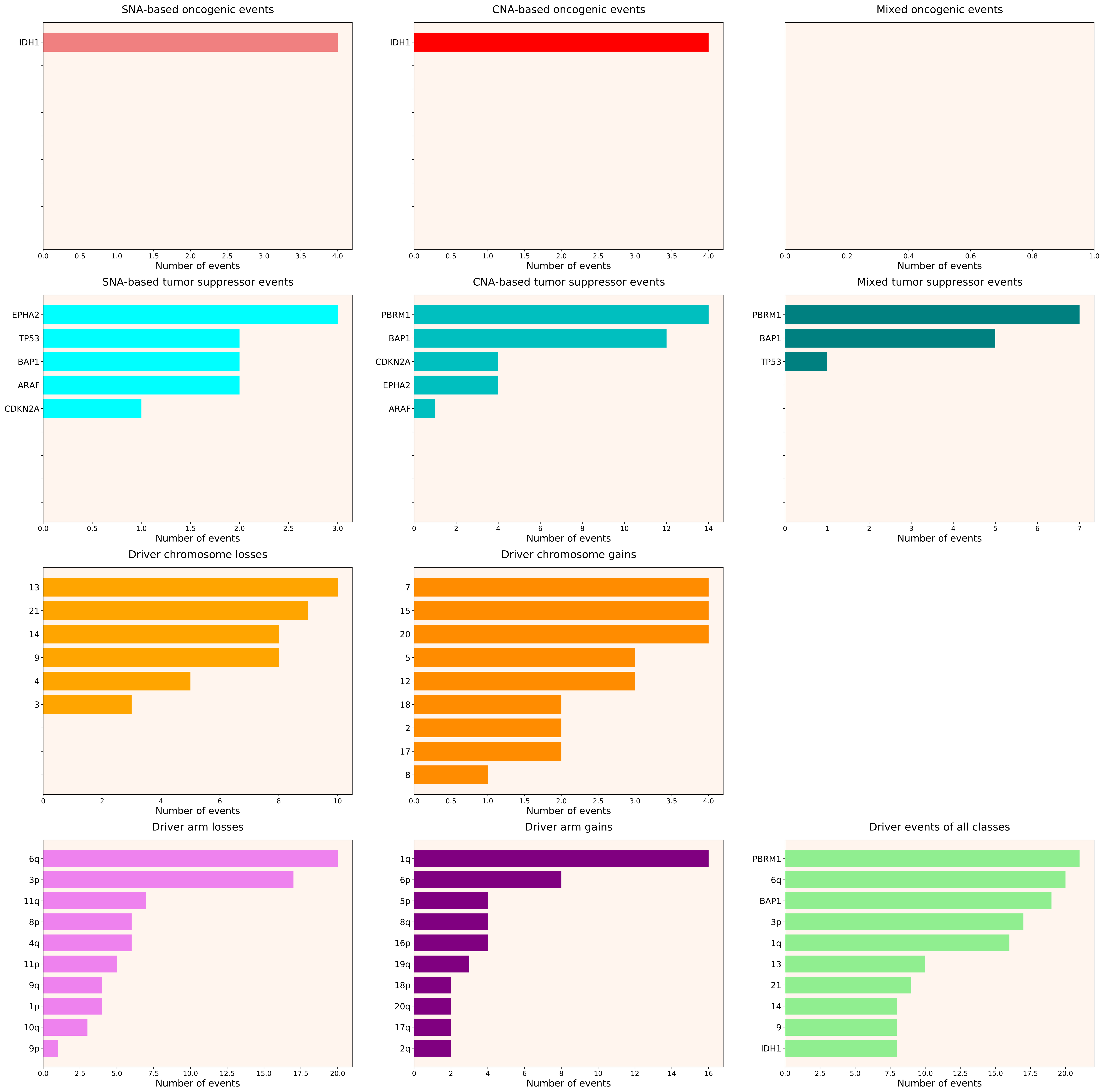

SNA-based oncogenic events
IDH1
Number of events
CNA-based oncogenic events
IDH1
Number of events
Mixed oncogenic events
Number of events
SNA-based tumor suppressor events
EPHA2
TP53
BAP1
ARAF
CDKN2A
Number of events
CNA-based tumor suppressor events
PBRM1
BAP1
CDKN2A
EPHA2
ARAF
Number of events
Mixed tumor suppressor events
PBRM1
BAP1
TP53
Number of events
Driver chromosome losses
13
21
14
9
4
3
Number of events
Driver chromosome gains
7
15
20
5
12
18
2
17
8
Number of events
Driver arm losses
6q
3p
11q
8p
4q
11p
9q
1p
10q
9p
Number of events
Driver arm gains
1q
6p
5p
8q
16p
19q
18p
20q
17q
2q
Number of events
Driver events of all classes
PBRM1
6q
BAP1
3p
1q
13
21
14
9
IDH1
Number of events
